# Long‐term effects of obesity on COVID‐19 patients discharged from hospital

**DOI:** 10.1002/iid3.522

**Published:** 2021-09-09

**Authors:** Luorui Shang, Li Wang, Fangyuan Zhou, Jinxiao Li, Yuhan Liu, Shenglan Yang

**Affiliations:** ^1^ Department of Integrated Traditional Chinese and Western Medicine, Union Hospital, Tongji Medical College Huazhong University of Science and Technology Wuhan China; ^2^ Department of Emergency Surgery, Union Hospital, Tongji Medical College Huazhong University of Science and Technology Wuhan China

**Keywords:** antibody titer, blood lipid, body mass index, COVID‐19, obesity, SARS‐CoV‐2

## Abstract

**Introduction:**

Obesity has been reported as a risk factor for COVID‐19 prognosis. However, the long‐term effects of obesity on patients discharged from the hospital are unclear, and the present study aims to address this issue.

**Methods:**

A cohort study was conducted using data from patients diagnosed with COVID‐19 who were discharged from Wuhan Union Hospital between February 20, 2020, and March 20, 2020. The 118 patients with COVID‐19 were divided into the non‐obesity group and the obesity group according to their body mass index (BMI). All the patients were invited to fill out a series of scales to assess cardiopulmonary function. Data on population baseline characteristics, clinical manifestations, laboratory examinations, chest computed tomography (CT), and lung function were collected and analyzed.

**Results:**

The clinical manifestations and pathological changes on CT images of obese patients were more serious after discharge than those of non‐obese patients. In addition, we found significant abnormalities in metabolic indicators such as blood lipids, uric acid, and liver function in obese patients. Most importantly, the antibody titer of COVID‐19 obese patients was inversely correlated with BMI.

**Conclusion:**

In the long term, obesity affects clinical manifestations, immune function and endocrine metabolism in patients discharged after recovering from COVID‐19.

## Graphical abstract

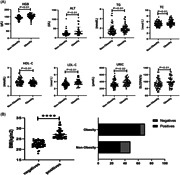

## 1 INTRODUCTION

Coronavirus disease 2019 (COVID‐19) first broke out in December 2019, and in a duration of slightly more than a year, it led to 83 million confirmed cases and 18 million globally. Obesity was reported as a risk factor for COVID‐19, which was strongly correlated with disease severity, admission to acute intensive care, death, and so on.^1^, ^2^, ^3^, ^4^ However, the long‐term effects of obesity on discharged COVID‐19 patients are unknown.

According to the World Health Organization, about 50% of the global population can be categorized as obese.^5^ Obesity leads to local and systemic chronic inflammation, which in turn results in various chronic immune and metabolic diseases, such as type 2 diabetes, cancer, atherosclerosis, and inflammatory bowel disease.^6^, ^7^ We hypothesized that obesity and obesity‐related comorbidities may adversely affect the recovery of discharged COVID‐19 patients.

To determine whether obesity has a long‐term impact on COVID‐19 recovery, we conducted a cohort study of 118 COVID‐19 patients admitted to and discharged from Wuhan Union Hospital, and compared the recovery of obese and nonobese patients considering their basic demographics, clinical manifestations, laboratory test results, chest CT scans, and pulmonary function test results.

## 2 MATERIALS AND METHODS

### 2.1 Ethics statement

All data obtained from the hospital medical records were anonymized before analysis. The study was approved by the Ethics Committee of Wuhan Union Hospital, Tongji Medical College of Huazhong University of Science and Technology. Written informed consent was provided by all survey participants before their enrollment (No. [2020]0526).

### 2.2 Study design and participants

This cohort study was conducted at Wuhan Union Hospital. We included 118 laboratory‐confirmed COVID‐19 patients who were discharged from Wuhan Union Hospital between February 20, 2020, and March 20, 2020. Based on their body mass index (BMI), the patients were divided into obese (>25 kg/m^2^) and nonobese (≤25 kg/m^2^) groups.^8^, ^9^, ^10^, ^11^ We excluded patients (1) who were pregnant women or nursing mothers, (2) with a mental disorder or a history of substance abuse or dependence, (3) who participated in other clinical trials in the previous 3 months, (4) who the researchers considered unsuitable to participate.

### 2.3 Procedures

We followed up on COVID‐19 patients discharged from Wuhan Union Hospital between February 20, 2020, and March 20, 2020. Follow‐up was conducted on January 1, 2021 solstice and January 20, 2021. Follow‐up appointments were made by telephone by trained staff, and the time and place of follow‐up were informed one week in advance. We collected data on patients' basic information, height, weight, and existing or new symptoms, and conducted physical examination and questionnaire surveys. The physical examination included routine blood test of venous blood samples, liver and kidney function tests, blood glucose and lipid measurements, lung function, test, chest computed tomography (CT), and antibody titer analysis. Serum‐IgG antibodies against the nucleocapsid protein of SARS‐CoV‐2 were measured using enzyme‐linked immunosorbent assay (ELISA). Questionnaire surveys included the Modified British Medical Research Council (mMRC) and Brog Dyspnoea Index Scale, which were used to describe breathing conditions under different levels of physical exercise. A higher score indicates greater difficulty in breathing.^12^, ^13^ In addition, we used the chest CT images and assigned lung pathology scores, with a score assigned to each lobe (total five lobes). The total CT scores were decided by consensus of both physicians using a randomized double‐blind method for each patient.^14^, ^15^, ^16^

### 2.4 Statistical analysis

Categorical variables were expressed as counts or percentages, and continuous variables were expressed as median and interquartile ranges. For normally distributed data, the independent‐group *t*‐test was used for comparison. The chi‐square test or Fischer's exact test was used to compare non‐normally distributed variables between the two groups. All analyses were performed in SPSS version 20.0 for Windows. *p *< .05 was considered to indicate statistical significance.

## 3 RESULTS

### 3.1 Demographic and clinical characteristics of COVID‐19 patients

Data from 118 recovered COVID‐19 patients (65 nonobese and 53 obese) were included in this study. Overall, the median age of all patients was 53 (44, 61) years. The median ages for non‐obese and obese patients were 57 (48, 62) and 51 (41, 58), respectively, with no significant difference. Male patients accounted for 41% of the patients (shown in Table 1). There was no significant difference in obesity prevalence considering sex. Smoking patients accounted for 15.4% of nonobese patients and 9.4% of obese patients. Obese patients had more underlying diseases, such as coronary heart disease (5.7% vs. 0%) and diabetes (5.6% vs. 4.6%) than nonobese patients. The most common residual symptoms after discharge include shortness of breath, fatigue, insomnia, joint pain, loss of smell, diarrhea, and constipation, which occurred in 40.6%, 36.4%, 42.4%, 33.1%, 7.6%, 3.4%, and 17.8% patients, respectively. Patients with mMRC score ≥1 accounted for 38.9% of the total number. The patients with Brog score ≥1 accounted for 39.8% of the total number of patients followed up. The median duration from symptom onset to follow‐up visit was 349 (346, 354) days and the median time from discharge to follow‐up visit was 323 (311, 330) days.

**Table 1:** Demographics and baseline characteristics of patients infected with SARS‐CoV‐2

	Number (%)	BMI (kg/m^2^)		
	Total (*n* = 118)	<25 (*n* = 65)	≥25 (*n* = 53)	*p* *
**Age, median (IQR), years**	53 (44–61)	57 (48–62)	51 (41–58)	.08
**Sex**				
Male	48 (41)	23 (35)	24 (45)	.27
Female	70 (59)	42 (65)	29 (55)	
**Smoking**	15 (12.7)	10 (15.4)	5 (9.4)	.35
**Hypertension**	20 (16.9)	12 (18.5)	8 (15.1)	.66
**Coronary heart disease**	3 (2.5)	0	3 (5.7)	.05
**Diabetes**	6 (5)	3 (4.6)	3 (5.6)	.80
**Signs and symptoms**				
Shortness of breath	48 (40.6)	26 (40)	22 (41.5)	.87
Fatigue	43 (36.4)	21 (32.3)	22 (41.5)	.31
Sleep difficulties	50 (42.4)	29 (44.6)	21 (39.6)	.59
Joint pain	39 (33.1)	20 (30.8)	19 (35.8)	.56
Smell disorder	9 (7.6)	5 (7.7)	4 (7.5)	.97
Diarrhea	4 (3.4)	2 (3.1)	2 (3.8)	.84
Constipation	21 (17.8)	16 (24.6)	5 (9.4)	.03
**mMRC score**				
**0**	72 (61.1)	41 (63.1)	31 (58.5)	.62
**≥1**	46 (38.9)	24 (36.9)	22 (41.5)	
**Brog score**				
**0**	71 (60.2)	39 (60)	32 (60.4)	.97
**≥1**	47 (39.8)	26 (40)	21 (39.6)	
**COVID‐19 clinical classification**				
Light	37 (31.4)	21 (56.8)	16 (43.2)	.43
Normal	41 (34.7)	18 (43.9)	23 (56.1)	.22
Severe	40 (33.9)	19 (47.5)	21 (52.5)	.12
**Time from discharge to follow‐up, days**	323 (311–330)	323 (311–330)	323 (313–329)	.99
**Time from symptom onset to follow‐up, days**	349 (346–354)	348 (342–353)	351 (346–356)	.33

* *p* values indicate differences between non‐obesity and obesity group patients. *p* < .05 was considered to indicate a statistically significant difference.

### 3.2 Comparison of laboratory parameters between nonobese and obese patients

**Figure 1:**
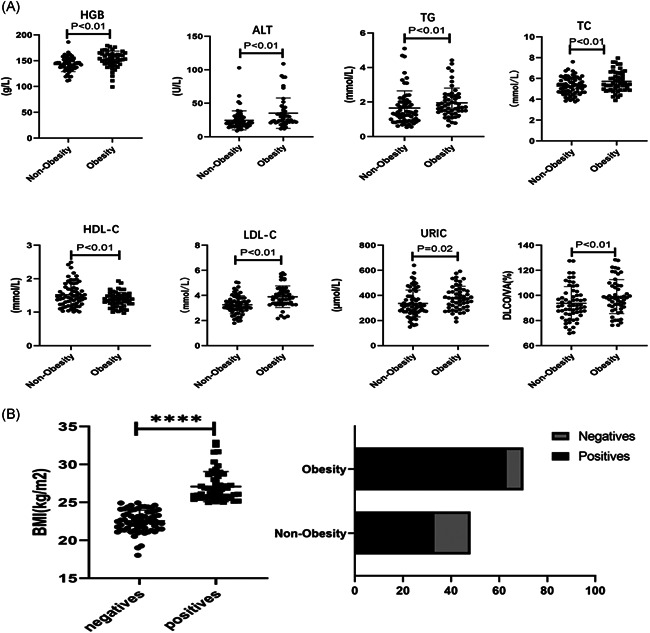
Blood test and biochemical examination results of obese and nonobese patients. (A) Blood test results, inflammation‐related laboratory findings, and lung function test results of nonobese and obese patients. (B) The correlation of body mass index with IgG antibody titer of the nonobese and obese patients. *p* < .05 was considered to indicate a statistically significant difference

Routine blood markers such as white blood cells (5.69 [4.83–6.63]) and lymphocytes (1.84 [1.55–2.19]) were slightly higher. The platelet count (212 [178–257]) slightly decreased in both groups. The increase in hemoglobin in obese patients (147 [138–158]) was significantly higher than that in nonobese patients (*p *< .01). Liver and kidney function test results showed that the increase in alanine aminotransferase level in obese patients (23[19–32]) was significantly higher than that in the nonobese patients (*p* < .01). Levels of aspartate aminotransferase (23 [20–28]), g‐glutamyltransferase (22 [15–35]), blood urea nitrogen (5.08 [4.44–5.88]), and creatinine (60.15 [50.10–69.33]) displayed no obvious differences between the two groups. Metabolic indexes such as uric acid (317.9 [269.63–392.83] vs. 375.9 [304.4–434.3]), triglyceride (1.33 [0.94–2.01] vs. 1.71 [1.41–2.32]), total cholesterol (5.26 [4.67–5.95] vs. 5.51 [4.97–6.48]), high density lipoprotein cholesterol (1.47 [1.24‐1.72] vs. 1.37 [1.25–1.51]) and low density lipoprotein cholesterol (3.18 [2.85–3.65] vs. 3.83 [3.36–4.34]) were all higher in obese patients than in the nonobese patients (shown in Table 2, Figure 1A).

**Table 2:** Comparison of laboratory parameters between nonobese patients and obese patients

		Median (IQR)	BMI (kg/m^2^)		
	Normal range	Total (*n* = 118)	<25 (*n* = 65)	≥25 (*n* = 53)	*p*
WBC (G/L)	3.5–9.5	5.69 (4.83–6.63)	5.5 (4.58–6.74)	5.77 (5.02–6.48)	.31
LY (G/L)	1.1–3.2	1.84 (1.55–2.19)	1.84 (1.44–2.19)	1.88 (1.56–2.22)	.25
HGB (g/L)	130–175	147 (138–158)	143 (135–152)	153 (143.5–163)	<.01
PLT (G/L)	125–350	212 (178–257)	215 (186–254)	208 (173–259)	.69
ALT (U/L)	5–40	23 (19–32)	21 (17–28)	26 (22–38)	<.01
AST (U/L)	8–40	23 (20–28)	23 (20–27)	24 (20–29)	.17
GGT (U/L)	10–60	22 (15–35)	18 (14–29)	26 (18–41)	.27
BUN (mmol/L)	2.9–8.2	5.08 (4.44–5.88)	4.94 (4.39–5.89)	5.37 (4.46–5.8)	.87
CREA (μmol/L)	41–81	60.15 (50.1–69.33)	59.35 (49.45–68.88)	60.2 (53.1–71.8)	.95
URIC (μmol/L)	155–357	338.75 (278.5–428.13)	317.9 (269.63–392.83)	375.9 (304.4–434.3)	.02
GLU (mmol/L)	3.9–6.1	5.5 (5.1–5.9)	5.5 (5.1–5.8)	5.7 (5.2–6.1)	.75
TG (mmol/L)	0–1.7	1.57 (1.14–2.16)	1.33 (0.94–2.01)	1.71 (1.41–2.32)	<.01
TC (mmol/L)	0–5.2	5.35 (4.79–6.09)	5.26 (4.67–5.95)	5.51 (4.97–6.48)	<.01
HDL‐C (mmol/L)	1.1–1.74	1.41 (1.24–1.6)	1.47 (1.24–1.72)	1.37 (1.25–1.51)	<.01
LDL‐C (mmol/L)	0–3.12	3.44 (3.03–4.04)	3.18 (2.85–3.65)	3.83 (3.36–4.34)	<.01

Abbreviations: ALT, alanine aminotransferase; AST, aspartate aminotransferase; BUN, blood urea nitrogen; COVID‐19, coronavirus disease 2019; CREA, creatinine; GGT, g‐glutamyltransferase; HDL‐C, high‐density lipoprotein; HGB, hemoglobin; IQR, interquartile range; LDL‐C, low‐density lipoprotein; LY, lymphocyte; PLT, platelet; TG, triglyceride; TC, total cholesterol; URIC, uric acid; WBC, white blood cell.

### 3.3 Lung function and chest CT at follow‐up according to BMI

After discharge, almost all patients had abnormal lung function. As showed in Table 3, obese patients had a higher proportion of forced expiratory volume in one second (FEV1; 9.4% vs. 8.3%), forced vital capacity (FVC; 1.9% vs. 1.7%), total lung capacity (TLC; 9.4% vs. 5.0%), residual volume (RV; 24.5% vs. 21.7%), and lung capacity (VC; 5.7% vs. 0%). However, the pulmonary diffusion function of obese patients was better than that of nonobese patients (7.5% vs. 11.7%; *p *< .05).

**Table 3:** Lung function and chest computed tomography (CT) findings at follow‐up

	Number (%)	BMI (kg/m^2^)		
	Total (*n* = 113)	<25 (*n* = 60)	≥25 (*n* = 53)	*p* *
**Lung function**				
FEV1 < 80%, % of predicted	10 (8.8)	5 (8.3)	5 (9.4)	.06
FVC < 80%, % of predicted	2 (1.8)	1 (1.7)	1 (1.9)	.31
FEV1/FVC < 70%	1 (0.9)	1 (1.7)	0	.44
TLC < 80%, % of predicted	8 (7.1)	3 (5.0)	5 (9.4)	.38
RV < 80%, % of predicted	26 (23)	13 (21.7)	13 (24.5)	.37
VC < 80%, % of predicted	3 (2.7)	0	3 (5.7)	.11
DLCO < 80%, % of predicted	29 (25.7)	18 (30)	11 (20.8)	.35
DLCO/VA < 80%	11 (9.7)	7 (11.7)	4 (7.5)	.03
**Chest CT**				
Number of patients	99	54	45	
GGO	16 (16.2)	6 (11.1)	10 (22.2)	.38
Irregular lines	47 (47.5)	19 (35.2)	28 (62.2)	.34
Bronchiectasis	15 (15.2)	5 (9.3)	10 (22.2)	.31
Nodular shadows	54 (54.5)	24 (44.4)	30 (66.7)	.83
Fibrosis	18 (18.2)	7 (12.9)	11 (24.4)	.54

Abbreviations: DLCO, diffusion capacity for carbon monoxide; FEV1, forced expiratory volume in one second; FVC, forced vital capacity; GGO, ground‐glass opacity; RV, residual volume; TLC, total lung capacity; VC, lung capacity.

* *p* values indicate differences between nonobese and obese patients. *p* < .05 was considered to indicate a statistically significant difference.

**Figure 2:**
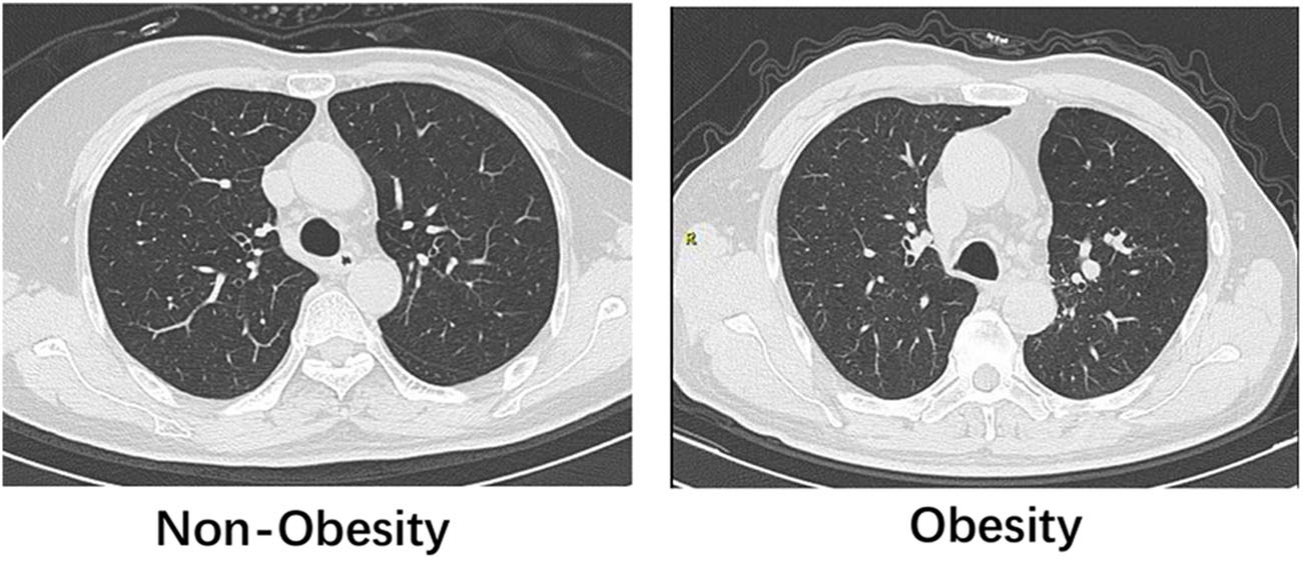
Chest computed tomography results of the nonobese and Obese patients

Ten months after discharge, most of the patients still had abnormal lung CT images. We compared the typical CT findings of obese and nonobese patients and found differences in the portion and extent of the lesions (shown in Figure 2). Obese patients, showed a higher proportion of ground‐glass opacity (22.2% vs. 11.1%), irregular lines (62.2% vs. 35.2%), bronchiectasis (22.2% vs. 9.3%), nodular shadows (66.7% vs. 44.4%) and fibrosis (24.4% vs. 12.9%) than non‐obese patients. However, these differences were not statistically significant (*p* > .05; shown in Table 3).

### 3.4 Correlation of BMI with IgG antibody titer

We compared IgG antibody in obese and nonobese patients, and found that the existence time of IgG antibody in obese patients was longer (Figure 1B).

## 4 DISCUSSION

To our knowledge, this is the first study to look at the long‐term effects of obesity on COVID‐19. We followed up patients after 12 months, from their discharge from the hospital to determine if obesity influenced their recovery from COVID‐19. We found no significant difference in recovery according to sex and age.^17^ Obese patients have comorbidities and consequently are at a higher risk of developing breathing problems. This may be because obese individuals have higher inflammatory cytokines than nonobese individuals.^18^

Obesity is linked to metabolic disorders,^19^ and we found that obese patients who recovered from COVID‐19 had an abnormal liver function, and higher uric acid, and lipid levels, than nonobese patients. We consider the following possible reasons: (1) A heightened inflammatory response caused by excessive release of cytokines after entry of SARS‐CoV‐2 in host cells can lead to oxidative stress. Impaired innate and acquired immune function, can lead to long‐term metabolic disorders. (2) Use of drugs during the acute infection period can result in abnormal liver function and elevated uric acid level, which does not recover to the normal level.^20^, ^21^ In addition, changes in dietary habits and lack of exercise may also lead to metabolic disorders in COVID‐19 patients after discharge.^22^, ^23^

Our data suggest that a large proportion of patients diffuse interstitial lung disease. Lung function impairment is higher in obese patients than in nonobese patients. Obesity is known to be associated with reduced lung function and adverse reactions to mechanical ventilation.^24^ This is attributed to the chronic pro‐inflammatory state caused by obesity, excessive oxidative stress, impaired immunity, and dysregulated cytokine signaling.^25^ Obesity can also alter the polarization of natural killer cells in COVID‐19.^26^ Physiologically, increased body weight is associated with decreased functional residual volume (FRC) and reduced expiratory reserve, which restricts expiratory flow and induces airway closure, thereby decreasing lung diffusion capacity in obese individuals.^27^ The residual pathological features seen in chest CT images of obese COVID‐19 patients are more likely because of the functional changes in adipose tissue in these patients, including decreased lipid storage capacity, increased expression of inflammatory factors, changes in secretion, adipose tissue hypoxia, and infiltration of macrophages in adipose tissue. These CT abnormalities were seen during the acute phase of the initial hospitalization; however, the current residual lesions did not fully recover during nearly 1 year of follow‐up.^28^, ^29^, ^30^

We found that serum IgG antibody levels in COVID‐19 patients were inversely correlated with BMI. It is reported that SARS‐CoV‐2 antibody titer is negatively correlated with levels of pulmonary inflammatory markers (SAA, CRP, and ferritin), leading to secretion of additional inflammatory markers, aggravating local and systemic inflammation, and resulting in B cell dysfunction.^31^, ^32^ Obesity can reduce the serum antibody response to influenza vaccine in young and old individuals^33^ and increase the secretion of autoimmune antibodies.

This study has several limitations. First, the sample size was insufficient, due to which we cannot rule out any statistical error. Second, the specific mechanism by which obesity leads to residual symptoms after discharge and abnormal laboratory results is still unclear. Furthermore, obesity in patients may be because of poor dietary habits and lifestyle after discharge, rather than development of obesity during hospitalization, which is what will be the topic of our future research.

In conclusion, our data suggest that obesity has a long‐term impact on COVID‐19 patients after hospital discharge and that follow‐up studies in larger populations for longer periods are necessary to fully understand the impact of obesity on COVID‐19 recovery.

## CONFLICT OF INTERESTS

The authors declare that there are no conflict of interests.

## AUTHOR CONTRIBUTIONS

Shenglan Yang had the idea and designed the study and had full access to all of the data in the study. Luorui Shang and Li Wang took responsibility for the integrity of the data and the accuracy of the data analysis. Yuhan Liu summarized the data. Shenglan Yang and Fangyuan Zhou took responsibility for project contact. Jinxiao Li was responsible for patient care and communication. All authors contributed to data acquisition or data interpretation, reviewed, and approved the final version.
